# Manipulating the Prion Protein Gene Sequence and Expression Levels with CRISPR/Cas9

**DOI:** 10.1371/journal.pone.0154604

**Published:** 2016-04-29

**Authors:** Lech Kaczmarczyk, Ylva Mende, Branko Zevnik, Walker S. Jackson

**Affiliations:** 1 German Center for Neurodegenerative Diseases (DZNE), Bonn, Germany; 2 Cologne Excellence Cluster on Cellular Stress Responses in Aging-associated Diseases (CECAD), Medical Faculty, University of Cologne, Cologne, Germany; Van Andel Institute, UNITED STATES

## Abstract

The mammalian prion protein (PrP, encoded by *Prnp*) is most infamous for its central role in prion diseases, invariably fatal neurodegenerative diseases affecting humans, food animals, and animals in the wild. However, PrP is also hypothesized to be an important receptor for toxic protein conformers in Alzheimer's disease, and is associated with other clinically relevant processes such as cancer and stroke. Thus, key insights into important clinical areas, as well as into understanding PrP functions in normal physiology, can be obtained from studying transgenic mouse models and cell culture systems. However, the *Prnp* locus is difficult to manipulate by homologous recombination, making modifications of the endogenous locus rarely attempted. Fortunately in recent years genome engineering technologies, like TALENs or CRISPR/Cas9 (CC9), have brought exceptional new possibilities for manipulating *Prnp*. Herein, we present our observations made during systematic experiments with the CC9 system targeting the endogenous mouse *Prnp* locus, to either modify sequences or to boost PrP expression using CC9-based synergistic activation mediators (SAMs). It is our hope that this information will aid and encourage researchers to implement gene-targeting techniques into their research program.

## Introduction

PrP is an extracellular, membrane-anchored glycoprotein, abundantly expressed in brain of a wide range of species, especially mammals [1]. The function of the physiological form of PrP, called the cellular form or PrP^C^ for historical reasons, is a matter of debate, but evidence exists for its role in synaptic plasticity [2–4], cell signaling [5], and neuroprotection [6–10], especially from stroke [11,12]. PrP^C^ can be subverted into neurodegenerative cascades via binding to toxic protein assemblies related to Alzheimer's disease [13–15]. In a group of rare neurological disorders known as prion diseases (PrDs) PrP is twisted into a toxic conformer–PrP^sc^–which is also an infectious agent (prion) [16,17]. Although rare, PrDs have many features in common with more prevalent neurodegenerative diseases, like Alzheimer’s or Parkinson’s diseases [18–20]. A remarkable feature of PrDs is that a single protein, PrP, can cause profoundly different diseases [21–23]. Thus, factors and mechanisms leading to differences in neurodegenerative diseases in general can be investigated by studying PrDs. Key insights into PrP biological functions were obtained from studies of transgenic mice expressing PrP mutants. For example, mouse studies conclusively showed that PrP^C^ is absolutely required for PrP^sc^ toxicity [24,25] and that sequence changes in PrP largely account for differences in vulnerability to prion infection [26,27]. However, mechanisms of prion replication and toxicity remain elusive, likely involving a complex interplay between physiological properties of individual cells, the amount and sequence of PrP expressed, and the nature and sequence of invading prions [28].

Much can be learned from creating mouse models expressing PrP variants. Such models can be engineered via gene-targeting of the endogenous gene locus (*Prnp*) to produce knock-in mice (KIs), or by allowing PrP expressing transgenes to integrate into random locations in the genome, resulting in random integration transgenic mice (RITs). An important advantage of the RIT approach, aside from the relative ease of the technique, is increased expression of PrP, accelerating the disease progression and exacerbating the effects of mutations [29–31] that are typically much milder when expressed from the endogenous location [22,32,33].

However, RITs are vulnerable to position effects leading to disparate spatial expression patterns in different lines, a confounder that is often ignored even though it likely contributes to phenotypic differences between different mutants expressed as RITs [34]. Unfortunately, manipulation of the endogenous *Prnp* locus in mice has been a challenge. The first attempt to knockout *Prnp* involved selecting, isolating, and screening of ~10.000 embryonic stem cell (ESC) clones by tedious Southern blotting [35,36]. Despite some significant improvements [32,37–39], the low efficiency of modifying *Prnp* in ESCs with homologous recombination (HR) has long thwarted efforts with this procedure, most likely due to a “closed” chromatin state of the locus [40–42]. To avoid this difficulty, most investigators continue to develop PrP transgenic mice with RITs, often driven by the moPrP.XhoI vector composed of fragments of *Prnp* from multiple sources [43–45]. New tools to manipulate endogenous *Prnp* were sorely needed to reduce the technological hurdles in creating *Prnp* KI mice.

**Fig 1:**
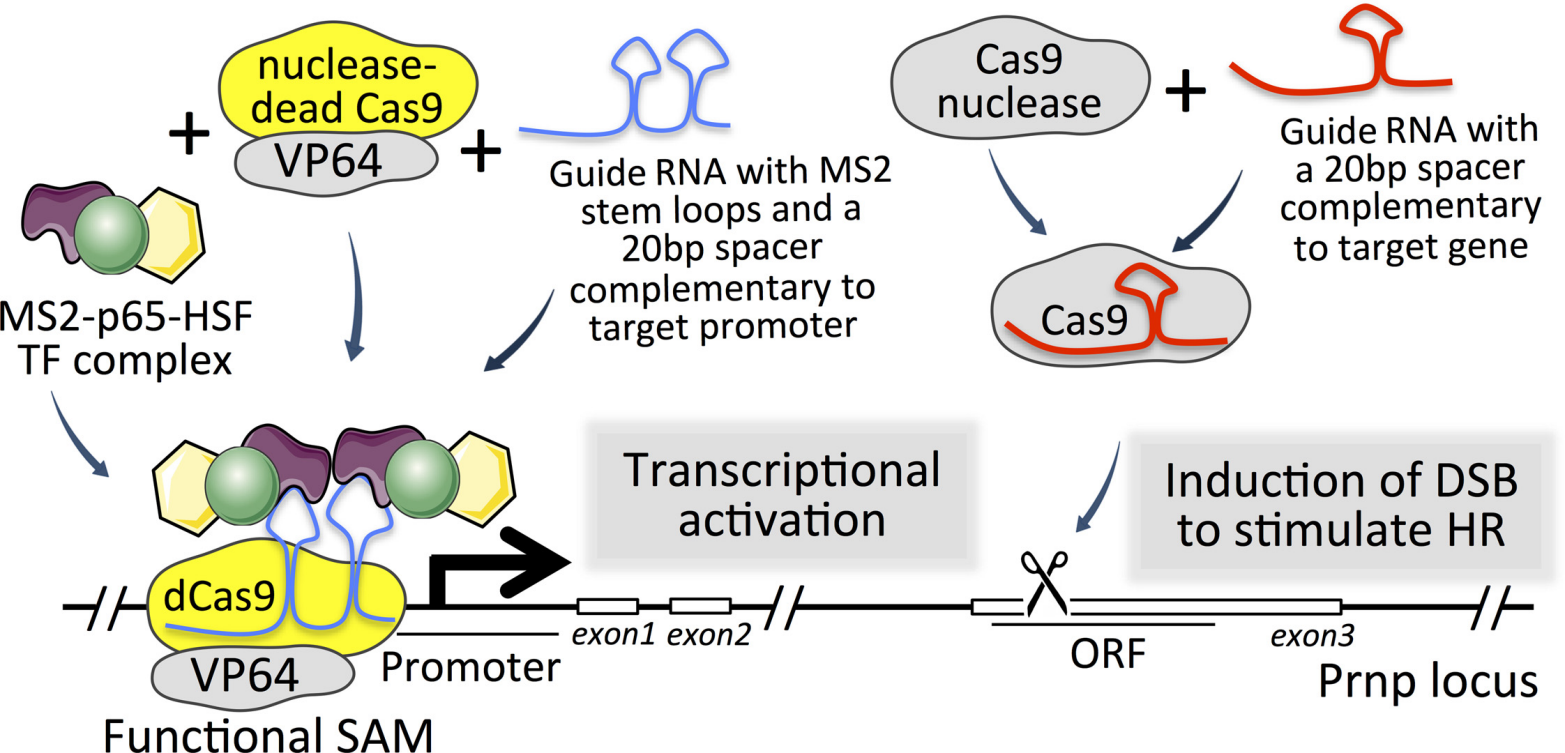
Two approaches involving CRISPR/Cas9 for gene manipulation. On the right, a Cas9 nuclease together with sgRNA (in red) induces a double strand break (DSB) in the DNA. Damage results in recruitment of DNA repair mechanisms, including homology-mediated repair (HDR), which can be harnessed to introduce and/or replace DNA fragments. On the left, a SAM complex consisting of catalytically inactive Cas9 (dCas9) fusion with VP64 activation domain and sgRNA2.0 (in blue) binds to a promoter region and stimulate gene expression. Transcription is further augmented by recruitment of a protein fusion comprising p-65 and heat shock factor 1 (HSF1) transcription factors and MS2 bacteriophage coat protein, which associate the complex to MS2 stem-loops in sgRNA2.0 [49,66].

Help came with the advent of powerful new genome engineering technologies, especially TALENs and CRISPR/Cas9 (CC9), which brought unprecedented precision and efficiency to targeted mutagenesis, making generation of gene KIs easier and faster [34,46–48]. Since the CC9 system is theoretically very simple to program to target specific locations by modifying the short single guide RNA (sgRNA) component (Fig 1), we tested its efficiency in stimulating HR in the *Prnp* locus in mouse ESCs. We evaluated several sgRNAs and identified some that were highly efficient in mutating ESCs and mouse fertilized oocytes. Furthermore, we evaluated several sgRNAs as components of SAM complexes (see Fig 1, left panel) for their efficiency in stimulating *Prnp* expression [49], obtaining up to 10-fold increases in mRNA and protein levels. Improved *Prnp* targeting will greatly facilitate production of more consistent disease models, and *Prnp*-directed SAMs can complement existing models by accelerating PrP expression ubiquitously, or in specific cell populations.

## Materials and Methods

### Ethics statement

Experiments were approved by the State Office of North Rhine-Westphalia, Department of Nature, Environment and Consumerism (LANUV NRW), approval number 84–02.04.2014.A372. All measures were taken to minimize the number of animals used, as well as to maximally reduce their discomfort and pain.

### Animals

Fertile male and female mice to generate fertilized oocytes were supplied by Charles River (France). Foster females and males for making them pseudopregnant (both RjHan:NMRI) were sourced from Janvier labs (France). Animals were kept in a 12 h light / 12 h dark photoperiod at 22°C (± 2°C) and a humidity of 55% (± 5%) with access to water and food ad libitum. Mice maintenance and handling was according to local government regulations and the Directive 2010/63/EU revising Directive 86/609/EEC).

### Vector generation

*Prnp* targeting vectors were generated using standard molecular biology techniques. A conventional *Prnp* KO (tagging) vector was constructed similar to a previous one [38] except that the HPRT mini-gene was blunt ligated into pWJPrP38 [32] opened by digestion with EagI and ClaI restriction endonucleases, thereby specifically removing PrP protein coding sequence but leaving the rest of exon 3 intact. KI targeting constructs were generated with a two-step process. First, a neomycin selection cassette flanked by Flp recombinase sites (Addgene plasmid #22687) was ligated into an engineered EcoRV site located 78bp upstream of *Prnp* exon 3 in the pWJPrP38 targeting vector template [32], creating pWJPrP 101. Second, PrP ORF variants (hamster and bank vole PrP variants, and PrP-EGFP fusion proteins) were ligated into the ClaI and EagI sites of pWJPrP101. Detailed descriptions of KI vectors will be included in reports describing the specific mouse lines. Spacer sequences to generate pX330 (Addgene plasmid #42230) and pX459 (Addgene plasmid #62988) derivatives for targeting *Prnp* locus were sourced as 5’ phosphorylated oligonucleotides (Sigma Aldrich), annealed through cycle of heating and slow cooling in 50mM NaCl, diluted to 100nM, and cloned into BbsI site. Vectors for SAMs were Addgene plasmids plasmids #61422 (MS2–p65–HSF1), #61423 (dCas9-VP-64), #61424 (sgRNA2.0 backbone) (see [49]). Spacers were cloned into sgRNA2.0 backbone same way as in case of pX330 and pX459.

### ES cell culturing, targeting and genotyping

Mouse V6.5 ES cells [50] were grown on gelatin-coated plates, on a confluent layer of PMEF-N fibroblasts (Millipore), at 37°C in 10% C0_2_, in DMEM containing high (4500 mg/L) glucose, 20% ES cell-qualified FCS (Millipore), 1x nucleosides (Millipore), 4 mM L-glutamine (Gibco), 1 mM Na-pyruvate (Gibco), 1x NEAA (Gibco), 1x Penicillin-Streptomycin (Gibco), 7 μl/L 2-mercaptoethanol and 1000 U/ml LIF (Millipore). ESCs were fed daily and split every 2–3 days. PMEF-N fibroblasts were grown in the same medium as ESCs, but containing standard FCS and no LIF and nucleosides. For electroporation, 5–10 x 10^6^ cells were used. ESCs were trypsinized, gently triturated to break cell clumps and suspended in ESC electroporation buffer (Millipore). Cells were electroporated using Gene Pulser Xcell (Biorad) set to 300V/250 μF. 40 μg of homology template and 10 μg of CC9 vectors were used per electroporation. DNA for electroporation was prepared by the following steps: 1) isolation from Stbl3 E. coli strain (Life technologies) using Endo Free MAXI Prep Kit (Qiagen), 2) overnight digestion of 100–200μg DNA with XhoI restriction enzyme (NEB) in 400μl reaction volume (1U of enzyme per μg of DNA, 3) precipitation by addition of 1ml 100% EtOH, 4) transferring the precipitated DNA using p1000 pipette to a new tube containing 1ml of 70% EtOH, 5) pelleting and resuspending in 100–200μl of ESC electroporation buffer (Millipore). After the pulse, cells were transferred into warm ESC medium and distributed into feeder-coated plates. Geneticin (Gibco, 350 μg/ml) selection was started after 18–24 hrs and continued for 3 days with daily change of the selection medium. Afterwards, colonies were grown for additional 4–6 days in ESC medium (until ready for harvesting), harvested with 1000 μl pipette, trypsinized and plated on gelatin and feeder-coated 24-well plates. Clones were expanded for 3–4 days, and then frozen, leaving small aliquots of cells further expanding for DNA isolation. For genotyping, DNA from confluent 24-well plates was isolated by lysis in buffer containing 10mM Tris-Cl pH 7.5, 10mM NaCl, 10 mM EDTA, 0.5% SDS and 1 mg/ml proteinase K, followed by EtOH precipitation. Long PCR to confirm genomic location were performed with LongAmp Taq polymerase (New England Biolabs), using 65°C annealing/extension temperature and 1 min/kb extension time. Used primer sequences are listed in S1 Table.

### Pronuclear injection (PNI) of CC9 components

Three days prior to PNI fifteen 3–4 weeks old C57BL/6NCrl females were injected intraperitoneal (i.p.) with 5 IU of PMSG (pregnant mare’s serum), followed by i.p. injection of 5 IU of hCG (human Choriongonadotropin) 48 h later. Females were subsequently mated 1:1 with 12 weeks old C57BL/6NCrl males. On the day of injection plug check was performed and females were sacrificed by cervical dislocation. Fertilized oocytes were collected upon preparing oviducts and rupturing the ampullae. Washing and incubation steps were performed as described elsewhere [51]. The injection mix (100 ng/μl Cas9 RNA, 50 ng/μl sgRNA, 20 ng/μl targeting construct DNA) was centrifuged for 10 min/4°C/9838 g and placed on ice prior to injection. Injections were directed to the male pronucleus. Constant pressure throughout the process led to injection of smaller amounts of injection mix into the cytoplasm. Unilateral embryo transfer into pseudopregnant foster females was either directly performed or when embryos reached the 2-cell stage. DNA from obtained founders was PCR amplified using 5’-ATCACCATCAAGCAGCACAC-3’ forward primer and 5’-CAACGTCAGTAGGACAATTTGA-3’ reverse primer, isolated from the gel and sequenced with the Sanger method using the forward primer.

### N2a cell maintenance, transfection and FACS sorting

N2a cells were cultured under the same conditions as MEFs. Confluent cells were transfected with combinations of vectors: MS2–p65–HSF1, dCas9-VP-64, and sgRNA2.0 containing *Prnp*-specific sgRNAs (*Prnp* SAM 1–9), with lipofectamine 3000 according to the manufacturer’s protocol, using respectively 15 μg / 15 μl / 15 μl of DNA/lipofectamine/P3000 reagent per 10 cm plate (or scaled proportionally). For fluorescence-activated cell sorting (FACS) experiments cells were harvested by trypsinization 24 h post transfection, suspended in MEF medium at a density of ~5x10^6^ cells/ml and kept on ice. EGFP-positive cells were enriched by FACS on FACSAria III cell sorter (Beckton Dickinson), using 530/30 emission filter for EGFP. Cell populations were gated as presented in S1 Fig. Sorted cell suspensions were chilled during sorting and (immediately after) spun down and snap-frozen (for immunoblotting) or lysed in RLT buffer (for sqRT-PCR).

### sqRT-PCR

Total RNA was purified with RNeasy Plus Mini Kit (Qiagen). Final RNA concentrations were adjusted to the value of the least concentrated sample. Reverse transcription was done with TaqMan Reverse Transcription Kit (ABI) using the manufacturer’s protocol, without addition of DTT, with a mixture of oligo d(T) and random hexamers for priming, and 0.5 μg of total RNA as template. 0.5 μl of obtained cDNA was used for RT-PCR reaction, using SYBR Green PCR Master Mix (ABI), in 384-well PCR plates (ABI) with a 7900HT Fast Real-Time PCR System (ABI). For each reaction, critical threshold cycle (Ct) value was determined using SDS 5.0 Software (ABI) and primer specificity was confirmed by melt curve analysis. Two independent primer pairs for *Prnp* were used, and the data were analyzed with the ΔΔCt method, using the average Ct values of HPRT and ß-actin (ACTB) for normalization. Primer sequences (5’-3’): Prnp1_F: GTCGCATCGGTGGCAGGACT, Prnp1_R: CAGCCAGTAGCCAAGGTTCGCC, Prnp2_F: ACATCTGAAGTATGGGACGC, Prnp2_R: TAGGGGTCTGCTTTGGAATC, HPRT_F: CTCTGGTAGATTGTCGCTTATC, HPRT_R: CTCTTAGATGCTGTTACTGATAG, ACTB_F: GTTACAGGAAGTCCCTCACC, ACTB_R: ACCAAAGCCTTCATACATCAAGT.

### Immunoblotting

Deep-frozen cell culture pellets were lysed in 2x LDS (lithium dodecyl sulfate) sample buffer containing 40 mM DTT. Lysates were forced through a 27G needle several times to cleave DNA and reduce viscosity, and then denatured at 80°C for 10 min prior to loading on 10%NuPAGE Novex midi gels (Life Technologies). Gels were run using MES [2-(N-morpholino) ethane sulfonic acid] running buffer at 160 V. Gels were electro-transferred to nitrocellulose membrane (Bio-Rad), submerged in transfer buffer (20% methanol, 25 mM Tris-Cl, 0.19 M glycine), using a Criterion transfer tank (BioRad), at 700 mA, for 70 minutes. Membranes were blocked for 20 min at RT in 5% powdered milk in PBST (PBS with 0.05% tween-20) and then incubated with primary antibody diluted in blocking buffer overnight at 4°C. Next, blots were washed 4x with PBST and incubated with secondary antibody for 30min at RT, followed by 5 PBST washes at RT and imaging with the Li-Cor Odyssey system. For re-probing, membranes were stripped for 10 min with Re-Blot solution (EMD-Millipore), followed by repeated blocking. Membranes were imaged with the Odyssey imaging system (LI-COR Biosciences). Primary antibodies: mouse anti-PrP SAF83 (dil. 1:2000, Cayman Chemical), mouse anti-α-tubulin (dil. 1:10000, Sigma Aldrich). Secondary antibody: donkey-anti-mouse IRDye 800CW (dil. 1:20000, Li-Cor).

### FACS analysis and immunocytochemistry on N2a cells

N2a cells were cultured and transfected as described above. For FACS analysis, to preserve cell surface PrP, cells were harvested without trypsin, by scraping in PBS (without Ca^2+^ and Mg^2+^). Prior to scraping, cells were incubated for 1h with SAF83 primary antibody (dil. 1:2000, Cayman Chemical), washed with PBS and incubated with Alexa 647-coupled goat-anti-mouse secondary antibody (Life Technologies). Prior to FACS, suspensions were filtered through 70μm mesh. Data were acquired using the FACS Canto II flow cytometer (BD Biosciences) and analyzed with FlowJo software v.10.1 (Tri Star, Inc.). For immunocytochemistry, cells were cultured on 8-well chambered coverglass (Nunc), stained identically as for FACS analysis, and imaged with LSM700 confocal microscope (Carl Zeiss).

### Statistical analysis

Gene targeting experiments results were assessed with Cochran-Mantel-Haenszel test for repeated tests of independence. In each case, the experiment was repeated at least three times, and at least eight colonies were analyzed per replicate. For sqRT-PCR in gene activation experiments, Kruskal-Wallis test was used, followed by Dunn’s multiple comparison post-test.

## Results

### Evaluation of CC9 sgRNAs for manipulating the *Prnp* locus

Prior to the discovery of CC9, we typically performed gene-targeting experiments using the double replacement or tag and exchange strategy [22,32,52,53]. This strategy involved 1) targeting (or “tagging”) the *Prnp* locus with a selectable marker (HPRT), and 2) re-targeting the locus, this time “exchanging” the tagged allele for an in vitro modified allele. The HPRT mini gene is an especially useful selectable feature: cells with HPRT activity are resistant to hypoxanthine-aminopterin-thymidine (HAT), whereas cells without HPRT activity are resistant to 6-thioguanine (6-TG) [32]. Using this strategy, our routine gene-targeting experiments were carried out on two to three replicates done on successive days. On each day, typically three 10 cm dishes were selected for each electroporation, occasionally resulting in a total of three to five positive clones, but often resulting in none. Since ESCs require fresh media nearly every day, at a cost of ~ two Euro per 10 cm dish, plus the time to perform the labor (which is invaluable), it became a significant investment to create these lines. Thus, there was much room for improvement.

**Fig 2:**
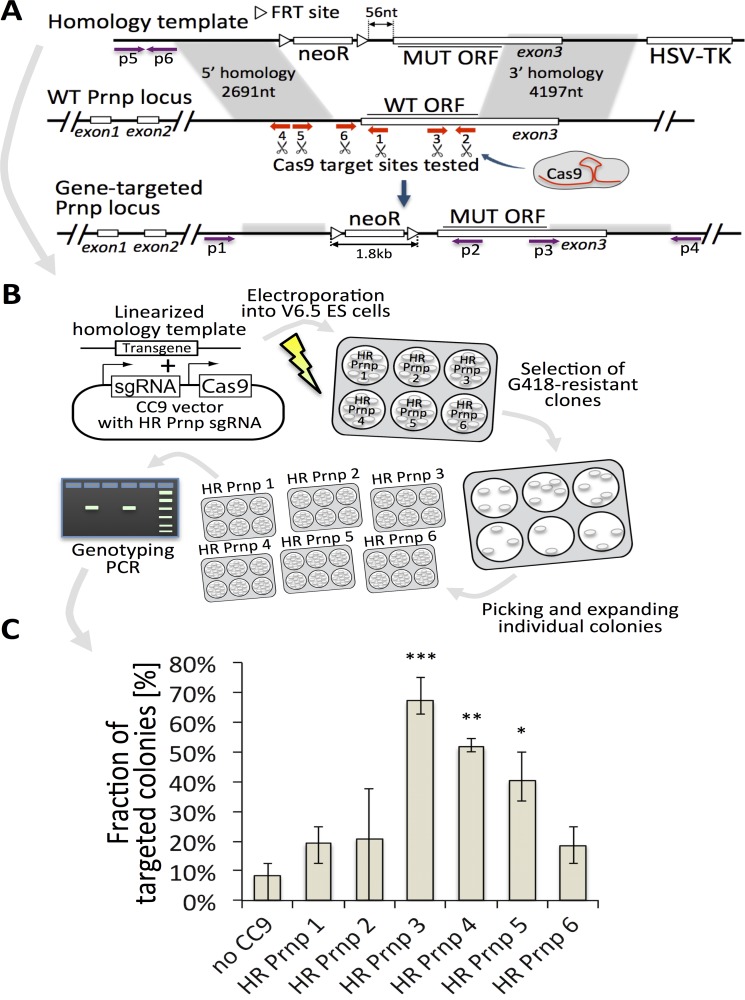
Evaluation of CRISPR/Cas9 for Prnp locus targeting. (**A**) Schematic gene knock-in into the mouse *Prnp* locus using homology vector used for all targeting experiments. sgRNA binding sited were marked with white arrows. Primer binding regions used for subsequent genotyping of recombined clones were marked with purple arrows and designated p1 through p6. NeoR–neomycin resistance gene; FRT–Flp recombinase recognition site for removal of selection cassette after successful gene targeting. Regions of homology were shaded in grey. (**B**) Schematic of the experimental workflow for sgRNA evaluation. In brief, cells were electroporated with a *Prnp* targeting vector only, or *Prnp* targeting vector and a CC9 encoding guides #1-#6. In each case, targeting efficiency was assessed by long PCRs spanning the homology arms. (**C**) Histogram of CC9-assisted targeting efficiency for each of the six tested sgRNA compared to “no CC9” control (first column). “*”—p<0.05; “**”—p < 0.01; “***”—p < 0.001; Cochran-Mantel-Haenszel test.

**Fig 3:**
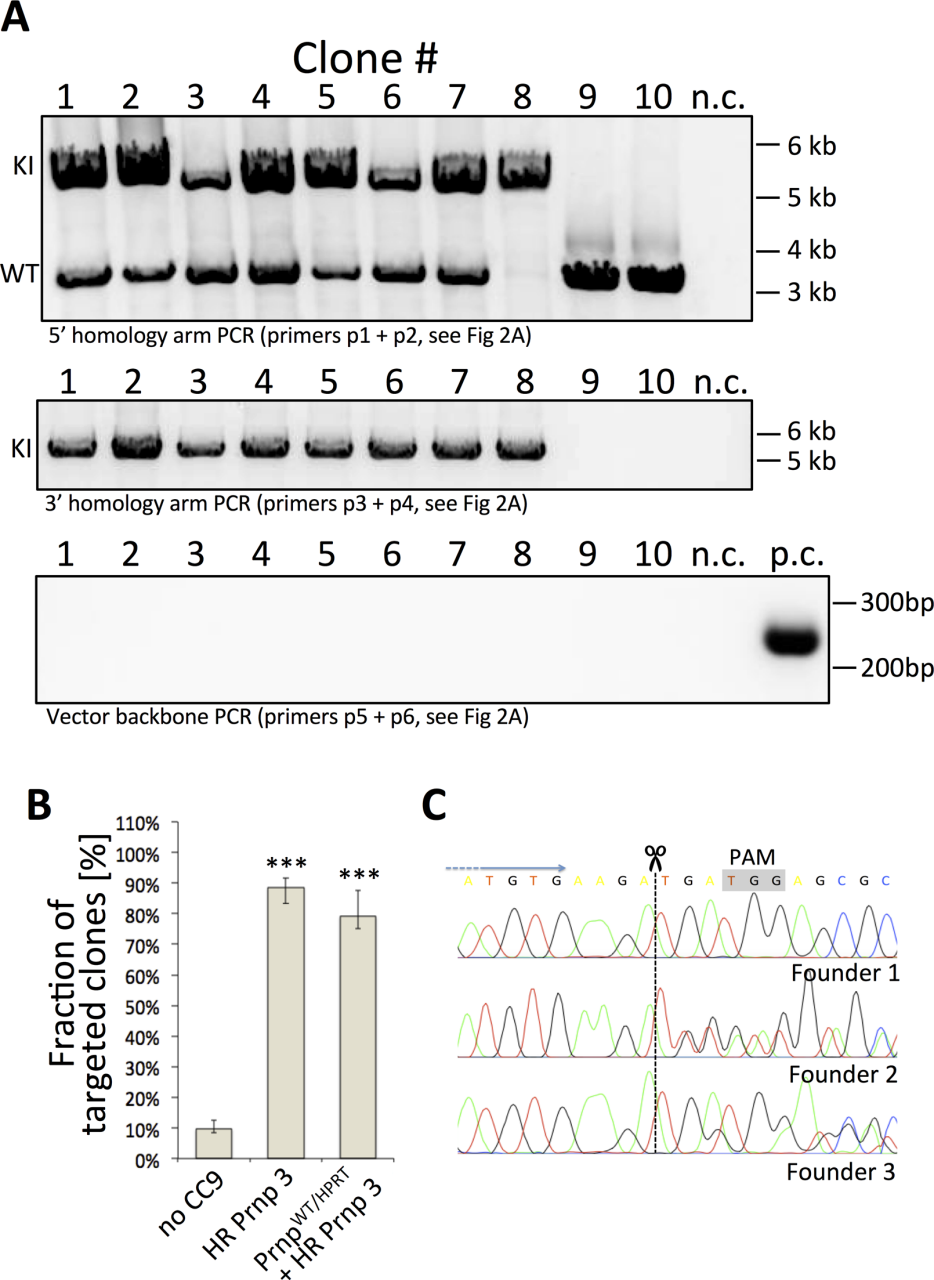
Targeting of the *Prnp* locus with CRISPR/Cas9. (**A**) Example PCR showing recombination of a 4kb long transgene into *Prnp* locus using HR *Prnp* 3 sgRNA; n.c.–negative control; p.c.–positive control; M—marker ladder. Reverse primer in the top panel anneals to both, WT and MUT sequences, and thereby can discriminate between homozygous and heterozygous knock-in; Clone #8 was confirmed to be homozygous. (**B**) Cumulative CC9 targeting efficiency assessment of with HR *Prnp* 3 sgRNA in unmodified V6.5 and V6.5 *Prnp*^(WT/HPRT)^ cells (containing only one copy of WT *Prnp* gene). “***”—p < 0.001; Cochran-Mantel-Haenszel test. (**C**) Fragments of sequencing chromatograms of DNA isolated from founder mice obtained by pronuclear injection of CC9 with HR *Prnp* 3 sgRNA. DNA sequencing of two out of three founders revealed a shift in electrophoregram corresponding to an in-del in the nuclease target site in one of the alleles. The relevant PAM sequence was shaded in gray.

For our initial assessment of *Prnp* gene targeting efficiency with CC9, we used the same homology template vector as with our numerous previous gene-targeting experiments, this time containing a 4kb transgene between the homology arms (Fig 2A). We designed six different sgRNAs and tested each separately (see Fig 2A and 2B and S2 Table). Spacer sequences were cloned into the pX459 CC9 vector, carrying expression cassettes encoding both hybrid sgRNA and spCas9-2A-Puro (Fig 2B) [54]. We envisioned using puromycin to select for cells expressing the puromycin resistance gene and thus the Cas9 nuclease (co-expressed by the spCas9-2A-Puro plasmid). Unfortunately, the exquisite toxicity of puromycin on ESCs is notoriously difficult to titrate and the conferred resistance from the spCas9-2A-Puro plasmid was insufficient to rescue our rapidly dividing ESCs. Therefore, our strategy relied on G418 selection of the neomycin resistance gene contained in the KI targeting construct. To facilitate the direct comparison of conventional tag and exchange targeting to CC9 directed HR, we created a heterozygous ESC line that is wild-type on one *Prnp* allele while the other allele had the PrP coding sequence (Fig 2A, "PrP ORF") replaced with an HPRT minigene (*Prnp*^WT/HPRT^, not shown). The targeting constructs included PrP sequences from different species, with numerous sequence differences from the mouse PrP coding sequence. As a result, the transgenes provided poor substrate for homologous recombination with the endogenous PrP coding sequence, and therefore the homology arms on the ends would be the only drivers of HR. Although the targeting constructs carried a negative selectable marker (HSV-TK), the drug it confers resistance to (gancyclovir) was not applied. Thereby, integration events were not selected against, creating a strategy analogous to the earliest gene-targeting experiments [35]. With this combination of targeting construct and either WT or heterozygous ESC lines, we were set to detect, within the same gene-targeting experiment, HR events dependent on CC9 compared to those from non-CC9 HR events. The latter would be expected to be enriched in events that exchanged the *Prnp*^HPRT^ allele and conferred resistance to 6-TG. When tested in WT or *Prnp*^WT/HPRT^ ESCs, three out of six guides led to significant improvement of the targeting efficacy as compared with the control experiments where no CC9 was used (Fig 2C). Interestingly, for experiments with *Prnp*^WT/HPRT^ cells, all G418-resistant clones were sensitive to 6-TG, indicating that the WT allele, and not the HPRT allele was replaced, and that HR facilitated by CC9 greatly exceeded HR without CC9. We selected guide #3 (HR *Prnp* 3, Fig 2C) for further experiments. Using the same homology template, we reproducibly obtained HR efficiencies exceeding those of random integration (the majority of G418-resistant cells were gene-targeted) (Fig 3A). Importantly, application of CC9 resulted in a nearly ten-fold increase in the proportion of clones that were positive (79.2±7.2% for HPRT/WT cells) and (88.6±4.6%%) for WT/WT cells) compared to experiments without CC9 (9.7±2.4%, Fig 3B).

Although we did not attempt to quantify the total number of clones, experiments with CC9 routinely yields far more colonies than our previous strategy (described at the beginning of this section), enabling us to reduce the overall number of cells needed for selection, typically obtaining many positive clones even when selecting on a single 6-well plate well. For example, instead of selecting for targeted clones on three 10 cm dishes of each electroporation, we now typically select on three 6-well plate wells, each typically yielding more than five positive clones. As the surface area of a 10 cm dish is ~150 cm^2^ and that for a 6-well plate well is 9 cm^2^, the shift from using a large number (six to nine) of 10 cm dishes to a small number of 6-well plate wells (three is enough to acquire three independent clones) has resulted in a significant reduction in culturing costs. Moreover, every experiment results in a large number of colonies, most of which are positive, allowing us to be highly selective when picking only morphologically perfect colonies, with high confidence that the gene-targeting attempt will not need to be repeated.

Encouraged by the high HR efficiency in ESCs, we then attempted to use CC9 with HR *Prnp* 3 to target transgenes of various sizes (3-7kb) into the *Prnp* locus in WT ES cells. Each clone was tested for correct recombination with two independent PCR assays, spanning 5’ and 3’ homology arms. We consistently obtained HR rates >80% for all clones analyzed (Table 1). In parallel, we attempted to induce HR by direct injection of all components into the pronucleus of fertilized mouse oocytes (PNI). To this end, we used the same homology template used in the initial screen of sgRNAs (4 kb fragment between homology arms). From a single PNI attempt, we obtained only three founder animals, with no homologous recombinants amongst them. Nevertheless, PCR sequencing of the CC9 target site revealed in-dels in two out of three founder lines, indicating CC9 with HR *Prnp* 3 is highly efficient *in vivo* (Fig 3C).

**Table 1:** Gene targeting efficiency into the *Prnp* locus using HR Prnp 3 spacer of four different targeting constructs containing variants of Bank Vole PrP and neomycin selection marker (FNF).

Targeting construct	Clones targeted/analyzed	Targeting efficiency
#1	20/22	91%
#2	18/19	95%
#3	16/20	80%
#4	18/19	95%

### Identification of CC9-based SAMs that activate *Prnp*

**Fig 4:**
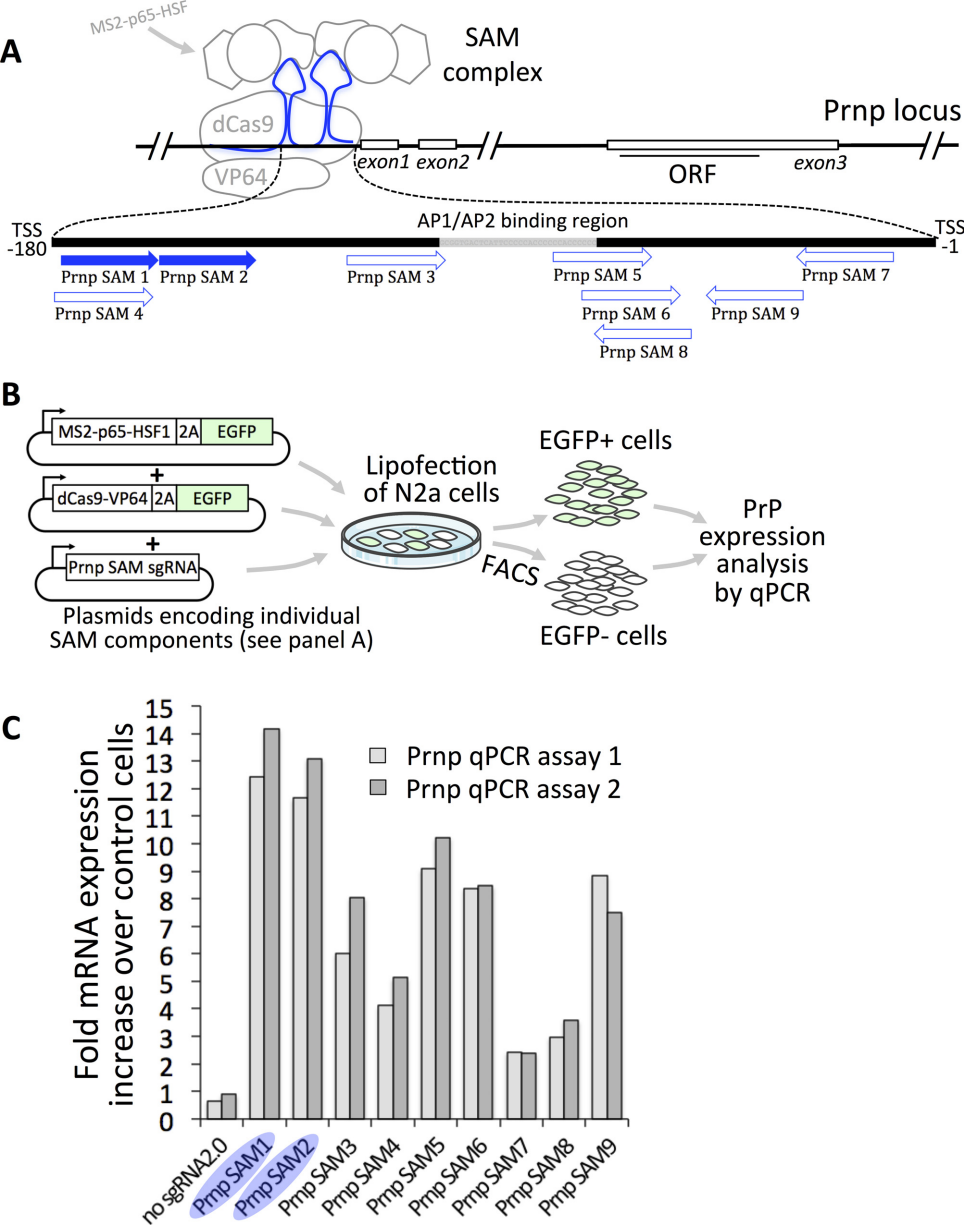
Evaluation of CRISPR/Cas9 SAMs directed to *Prnp*. (**A**) Relative positions of SAM sgRNA spacers with respect to the *Prnp* promoter region. Binding sites for AP1 and AP2 transcription factors were highlighted. (**B**) Schematic of the experimental workflow for SAM sgRNA evaluation. (**C**) Preliminary screening of SAM guides; sqRT PCR histogram shows increases in PrP protein levels for each individual SAM sgRNA tested, normalized to untransfected control levels (set to 1); assays #1 and #2 refer to independent primer pairs directed (respectively) to 5’- and 3’-proximal region of PrP mRNA.

**Fig 5:**
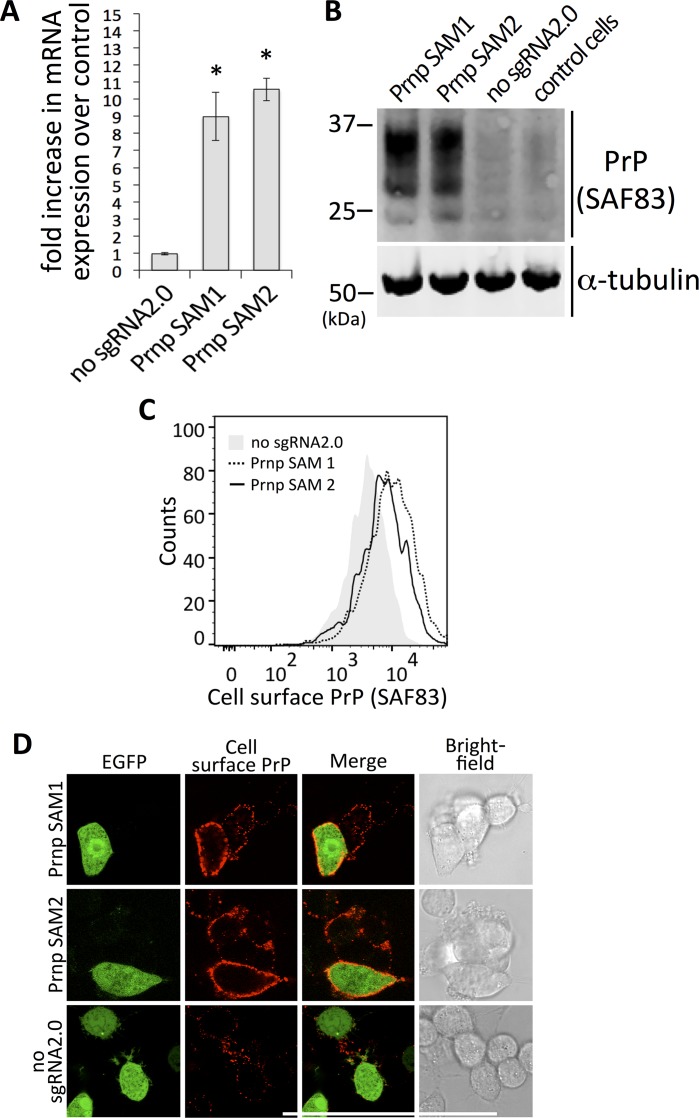
CRIPSR/Cas9 SAMs-directed activation of PrP expression. (**A**) sqRT PCR evaluation of Prnp SAM 1 and 2; “*”—p<0.001; Kruskal-Wallis test. (**B**) Immunoblot (SAF83) assessment of PrP levels after SAM activation using sgRNAs #1 and #2. (**C**) FACS of N2a cells transfected with Prnp-directed SAMs; histograms shows increased mean fluorescence intensity (MFI) corresponding to cell surface PrP (SAF83) on cells transfected with Prnp SAM 1 and 2 (dotted and solid line, respectively) as compared with MFI of “no_sgRNA2.0” control (in grey). (**D**) Immunostaining of N2a cells transfected with Prnp-directed SAMs; PrP levels on cells transfected with functional SAMs were increased as compared to untransfected (EGFP negative) cells in the same FOV. In case of “no sgRNA2.0” control, no differences between transfected and untransfected cells with respect to PrP staining were observed.

In addition to its powerful ability to modify genomic sequences, the CC9 system can also be employed to induce [49,55] or repress [55,56] gene expression. Multiple strategies have been successfully demonstrated but they all share the features of using a Cas9 derivative with the nuclease activity inactivated (dead Cas9 or dCas9) and further modified to recruit transcriptional transactivation components, such as through inclusion of the very robust VP64 transactivation domain (Fig 1). Importantly, the dCas9-VP64 fusion protein is directed to specific genomic locations with sgRNAs, just like for experiments with nuclease active Cas9. In the system we employed, the guide RNAs also included an extra pair of stem loops that recruit an additional transactivation driving fusion protein, MS2-p65-HSF. To determine if we could boost mouse *Prnp* expression with this system, we designed nine sgRNA spacers targeting various regions upstream of the *Prnp* transcription start site (TSS) (Fig 4A), with the aid of an online SAM sgRNA specificity prediction tool (http://sam.genome-engineering.org/database/) and sgRNA scoring algorithm [57], http://www.broadinstitute.org/rnai/public/analysis-tools/sgrna-design-v1). Sequences and scores of individual guides used in this study are included in S3 Table. Since ESCs divide ~ three times per day, we used a murine N2a neuroblastoma cell line since they divide far less frequently and will allow for the CC9 components to be expressed and then potentially regulate *Prnp*. N2A cells were transfected with combinations of vectors encoding 1) dCas9-VP64 fusion, 2) MS2–p65–HSF1 fusion protein and 3) sgRNA2.0 backbone containing MS2 stem loops and test gRNA spacers (Fig 4B) [49]. Components 1 and 2 also contained an EGFP reporter, enabling us to enrich for transfected cells by FACS (S1 Fig). While all tested sgRNAs led to an increase in PrP RNA levels, as determined by sqRT-PCR, sgRNAs #1 and #2 provided the greatest enhancement (Fig 4C). These two guides were further evaluated in three additional transfection experiments, inducing an average fold increase in expression of 9.0±1.4 for *Prnp* SAM 1 and 10.56±0.65 for *Prnp* SAM 2, but no increase was observed if the sgRNA was omitted (0.97±0.07, Fig 5A). Increased levels of PrP were detected on immunoblots indicating the extra mRNAs were functional (Fig 5B).

### Stimulation of *Prnp* with CC9 SAMs leads to overexpression of extracellular membrane-anchored PrP

PrP^c^ functions in health and disease depend on its correct localization in the membrane, attached to the outside surface by a GPI-anchor [7,58]. We therefore performed extracellular antibody labeling experiments, followed by FACS and immunocytochemistry (ICC) analyses, to determine if boosting PrP levels with SAMs leads to overproduction of correctly localized PrP. FACS analysis showed higher mean fluorescent intensity (MFI) for cells transfected with functional SAMs compared to cells transfected similarly but without the sgRNA, indicating that CC9 induced *Prnp* transactivation leads to increased levels of extracellular PrP (Fig 5C). Similarly, ICC experiments revealed increased extracellular PrP levels on cells transfected with functional SAMs compared to untransfected (EGFP negative) cells in the same field of view. In contrast, PrP staining in cells treated with a transfection mix lacking sgRNA appeared identical to that in non-transfected cells (Fig 5D), again indicating the increased *Prnp* activity directed by CC9 transactivation lead to increased amounts of properly localized PrP.

## Discussion

Pathological conformers of PrP cause a wide range of fatal and infectious neurodegenerative diseases known as transmissible spongiform encephalopathies (TSEs). Although the infectious component of these diseases are unusual in that the transmissible agent primarily consists of protein (hypothesized to lack disease encoding nucleic acids), their neurodegenerative component resembles more common neurodegenerative diseases, like AD or PD [59,60]. This makes modeling TSEs in biological systems invaluable for studying neurodegenerative processes, especially as different perturbations to the same protein can diverge into profoundly different pathologies [22]. For example, two of the most common mutations linked to familial prion diseases caused by single codon substitutions in *Prnp*, E200K and D178N, cause Creutzfeldt-Jakob disease (CJD) and fatal familial insomnia (FFI). The E200K variant primarily targets the neocortex and thus primarily causes a cognitive disease, whereas the D178N variant primarily targets the thalamus and disrupts sleep homeostasis and autonomic nervous system functions [21,22]. Such selective vulnerability of neurodegenerative diseases is by no means an exclusive characteristic of TSEs [21,61]. Indeed, in Alzheimer’s disease hippocampal and entorhinal cortical areas are primarily targeted, in Huntington’s disease the striatum is most severely affected, and in Parkinson’s disease the *substantia nigra* in the midbrain is notoriously damaged. Identifying the specific factors enabling different mutants of PrP (a protein ubiquitously expressed in CNS [53]) to target different brain regions and cause different diseases might improve our understanding of general mechanisms of more common neurodegenerative diseases. However, experiments of mouse models of familial PrDs show that, not only the mutations themselves, but also the genomic context of the transgene profoundly affects the disease phenotype [22]. For example, the mutation causing FFI does not always cause a neurodegenerative phenotype when expressed as a RIT, even when the gene product is present several fold higher than when expressed from the endogenous *Prnp* locus, a context in which the mutant gene is toxic [29,32]. A recently proposed but widely cited hypothesis postulates that miRNA levels can be modified by changes in their RNA targets [34,62,63]. The change in a miRNA's level in turn influences the levels of its other target RNAs, creating a complex gene regulatory system referred to as competing endogenous RNAs. It is conceivable that ectopically expressing *Prnp* derived transgenes containing *Prnp* derived regulatory elements will affect the expression of other genes sharing the interactome (e.g. miRNA binding sites) of these regions via a competing endogenous RNA mechanism. In this case the mouse phenotype would be influenced by destabilization of gene regulatory mechanisms, in addition to the protein-encoding component of the transgene.

The subtle (yet crucial for the disease) differences between *Prnp* knock-in and PrP RIT mice are likely to increase the demand for more precise *Prnp* knock-in models, and the tools for manipulating *Prnp in vivo*. With its versatility and ease of use, the CC9 system is a good choice, and was previously used to generate *Prnp* knockout cells, using a sgRNA targeting nucleotide 105 of the ORF [64]. A site close to the translation start site was targeted to prevent expression of potentially toxic truncated variants and assure a complete knockout. In our approach, we used CC9 to stimulate HR in *Prnp* to improve gene-targeting efficiency to create knock-ins. We searched guide sequences targeting the endogenous ORF that differed from the ORF in our targeting constructs (containing PrP coding sequences from species other than mouse) to avoid cleaving the homology templates used. We do not underestimate the importance of designing guides that are unlikely to target unintended sites. Fortunately, when generating gene-targeted mice nearly all such mutations can be breed away by crossings with wild-type mice. It is always a good idea, even when using conventional gene-targeting strategies, to breed new lines to wild-type mice for multiple generations to reduce (if not eliminate) spontaneous mutations acquired in culture. Therefore, doing so for ESCs exposed to CC9 does not equate to an extra burden of work.

We also found it very straightforward to employ SAM variants of CC9 to induce activity of native *Prnp*, with all of its regulatory elements, introns, and untranslated regions intact. In contrast to our sequence manipulation experiments, our *Prnp* activation experiments targeted regions upstream of the translation start site, showing the most distal guides to be most efficient (Fig 4). These were located upstream of AP1/AP2 transcription factor binding sites, which are likely critical elements of the *Prnp* promoter region [65].

The common feature of all CC9-related methods is that their specificity and efficiency depend on the sgRNA, ideally leading the Cas9 complex specifically to the intended target. Thus, individual sgRNAs differ in their performance. Despite continuous advances in design and quality control [66,67], the incomplete predictability of the CC9 system enforces two important points: i) restrictive consideration of off-target effects in individual experiments and ii) laborious quality control procedures [68]. Here, we observed variable performance of guide RNAs, despite using design tools [57,69]. Therefore, screening of multiple guides for a particular application may improve the likelihood of the successful application of the various CC9 techniques. For experiments where off target effects need to be ruled out but for which off target changes can not be bred away (e.g. in SAM experiments), employing multiple guides targeting non-overlapping sites will assure the effects observed are not due to off target artifacts, analogous to how many RNAi experiments are currently performed. Noteworthy, at the time of writing this manuscript a novel Cas9—Cas9 High Fidelity (HF)—mutant was described [70], reported to have comparable efficiency, but much reduced off-target activity compared to its predecessor.

Until recently, the efficiency of HR in *Prnp* was a major bottleneck for generating knock-in mouse lines. Surprisingly, in our hands, CC9 stimulated HR of transgenes specifically into the *Prnp* locus at levels exceeding that of random integration. Therefore, applying CC9 methods in cell culture experiments employing stable transfections will likely be more efficient than conventional random integration methods, with the added benefit that each variant will be targeted to the same locus. This will also facilitate the generation of *Prnp* knock-in mouse models by direct injection or electroporation of the CC9 components into fertilized oocytes [48,71], thereby bypassing the ESC culturing step, reducing costs, time and the stress when ESCs fail to contribute to the germ-line of chimeras. Although our PNI attempts to replace a nearly 1 kb segment of DNA with a completely different 1 kb sequence did not produce homologous recombinants, at the time very few such attempts of inducing HR of such large transgenes had been reported, limited to those inserting short reporters or tags [72,73]. Moreover, we obtained only three founder animals–a small number compared to what was screened in other studies. Nonetheless, sequencing of *Prnp* from the founders revealed that two out of three contained an in-del in this region (near nucleotide 610 of the ORF), which confirmed that the CC9 system is also highly efficient at targeting *Prnp in vivo* (Fig 3C). It is conceivable that we would also produce homologous recombinants if we screened more founder mice. Nonetheless, using HR *Prnp* 3 sgRNA presented here, we have successfully generated more then ten *Prnp* knock-in ESC lines, each with multiple clones. Five of these clones were used for blastocyst injection, giving rise to knock-in mouse lines and making further zygote injections redundant. Noteworthy, our HR *Prnp* 3 guide targets codon 204 in close proximity of the critical codons 199 and 209 (200 and 210 in human sequence), making it potentially useful to generate CJD-related mutations (E200K, V210I) using short oligonucleotides as HR templates.

In spite of the unpredictable expression patterns of RITs, they are very useful when higher than normal expression levels are desired. This proved invaluable for studying the effects of mutations on prion replication in vivo and for accelerating the disease phenotypes in prion infection [43,74–77]. The benefits also include significantly shorter experiment time and reduced costs related to mouse caging. However, uncontrolled overexpression, especially for transgenes driven by artificial promoters, can lead to unintended problems. A startling high proportion of proteins expressed at natural levels fail to pass endogenous quality control mechanisms [78]. This is especially problematic for PrP and other neurodegenerative disease related proteins due to their intrinsic tendency for aggregation. When ectopically expressed in cell types poorly equipped to process them, this would likely burden intrinsic protein quality control and degradation systems in an unnatural way, one not experienced in human disease.

As an alternative we propose amplification of endogenous *Prnp* gene expression using targeted transcriptional activators, such as SAMs [49]. This is not trivial, as membrane anchoring of PrP is preceded by several energetically expensive post-translational modifications, that include cleavage of the N-terminal endoplasmic reticulum targeting signal peptide, addition and maturation of two independent oligosaccharide chains, formation of a disulphide bridge, and attachment of the GPI membrane anchor [79]. It is conceivable that abnormally high expression of a protein (where it would normally be absent or present at much lower levels) will result in these mechanisms being inefficient, and for inadequately processed PrP be retained in the ER or targeted for degradation. However, our results suggest that a significant amount of PrP overexpressed upon stimulation with SAMs is membrane localized. We did not test the SAM approach in cell types other than N2a, and therefore cannot rule out the possibility that the ability of the cells to correctly process overexpressed PrP is cell type-dependent. Nonetheless, in contrast to ectopic overexpression systems, or RITs, our system is likely to be more predictable and easy to test and fine-tune (e.g. by titrating the amounts of donor vectors) before embarking on *in vivo* experiments.

In combination with currently available nucleic acids delivery methods (improved virus-based systems, *in vivo* electroporation) an approach involving SAMs may combine the advantages of knock-ins (proper spatial expression pattern) with the main experimental advantage of RITs, allowing one to boost PrP levels in a specific cell type and time (determined by employing appropriate promoters and the delivery time of the vectors). For mouse experiments, it would accelerate disease progression and thereby shorten experimental read-out time. For cell culture experiments, it should increase the efficiency of *in cyto* prion infections, as well as exacerbate the effects of prion protein in cell culture studies [80–82] and in organotypic slice paradigms [83–85]. SAM sgRNA2.0 vectors that we describe here (the plasmids for episomal expression and lentiviral transduction encoding the relevant sgRNAs are available from Addgene) might serve as a starting point for such experiments, and validated guide sequences may provide a foundation for generating CC9-based PrP activators for other applications. The CC9 system is a very powerful, and yet easy to implement, platform that we hope will be widely adopted in the prion disease field.

## Supporting Information

S1 Fig FACS sorting of N2a cells. (PDF)

S1 Table Primer sequences used for genotyping ES cell clones. (PDF)

S2 Table CC9 guide sequences used to stimulate HR in Prnp locus. (PDF)

S3 Table CC9 guide sequences used to generate SAM sgRNAs for activating Prnp gene. (PDF)

S4 Table Gene targeting experiments and SAM screening–raw data. (PDF)

## Abbreviations

Cas9: CRISPR-associated system nuclease 9
CC9: CRISPR/Cas9
CRISPR: clustered regularly interspaced short palindromic repeats
DSB: double strand break
ESCs: embryonic stem cells
HR: homologous recombination
NHEJ: non-homologous end joining
*Prnp*: prion protein gene
PrP: prion protein
RIT: random integration transgenic
SAM(s): synergistic activation mediator(s)
sgRNA: short guide RNA
TALENs: transcription activator-like effector nucleases
ZFNs: zinc finger nucleases

## References

[1] van Rheede T, Smolenaars MM, Madsen O, de Jong WW (2003) Molecular evolution of the mammalian prion protein. Mol Biol Evol 20: 111–121. 12519913 10.1093/molbev/msg014

[2] Shorter J, Lindquist S (2005) Prions as adaptive conduits of memory and inheritance. Nat Rev Genet 6: 435–450. 15931169 10.1038/nrg1616

[3] Caiati MD, Safiulina VF, Fattorini G, Sivakumaran S, Legname G, et al (2013) PrPC controls via protein kinase A the direction of synaptic plasticity in the immature hippocampus. J Neurosci 33: 2973–2983. 10.1523/JNEUROSCI.4149-12.2013 23407955 PMC6619229

[4] Maglio LE, Perez MF, Martins VR, Brentani RR, Ramirez OA (2004) Hippocampal synaptic plasticity in mice devoid of cellular prion protein. Brain Res Mol Brain Res 131: 58–64. 15530652 10.1016/j.molbrainres.2004.08.004

[5] Linden R, Martins VR, Prado MA, Cammarota M, Izquierdo I, et al (2008) Physiology of the prion protein. Physiol Rev 88: 673–728. 10.1152/physrev.00007.2007 18391177

[6] Roucou X, Gains M, LeBlanc AC (2004) Neuroprotective functions of prion protein. J Neurosci Res 75: 153–161. 14705136 10.1002/jnr.10864

[7] Westergard L, Christensen HM, Harris DA (2007) The cellular prion protein (PrP(C)): its physiological function and role in disease. Biochim Biophys Acta 1772: 629–644. 17451912 10.1016/j.bbadis.2007.02.011 PMC1986710

[8] Chiarini LB, Freitas AR, Zanata SM, Brentani RR, Martins VR, et al (2002) Cellular prion protein transduces neuroprotective signals. EMBO J 21: 3317–3326. 12093733 10.1093/emboj/cdf324 PMC125390

[9] Nishida N, Tremblay P, Sugimoto T, Shigematsu K, Shirabe S, et al (1999) A mouse prion protein transgene rescues mice deficient for the prion protein gene from purkinje cell degeneration and demyelination. Lab Invest 79: 689–697. 10378511

[10] Bremer J, Baumann F, Tiberi C, Wessig C, Fischer H, et al (2010) Axonal prion protein is required for peripheral myelin maintenance. Nat Neurosci 13: 310–318. 10.1038/nn.2483 20098419

[11] Weise J, Sandau R, Schwarting S, Crome O, Wrede A, et al (2006) Deletion of cellular prion protein results in reduced Akt activation, enhanced postischemic caspase-3 activation, and exacerbation of ischemic brain injury. Stroke 37: 1296–1300. 16574930 10.1161/01.STR.0000217262.03192.d4

[12] Steele AD, Zhou Z, Jackson WS, Zhu C, Auluck P, et al (2009) Context dependent neuroprotective properties of prion protein (PrP). Prion 3: 240–249. 19901559 10.4161/pri.3.4.10135 PMC2807698

[13] Lauren J, Gimbel DA, Nygaard HB, Gilbert JW, Strittmatter SM (2009) Cellular prion protein mediates impairment of synaptic plasticity by amyloid-beta oligomers. Nature 457: 1128–1132. 10.1038/nature07761 19242475 PMC2748841

[14] Nygaard HB, Strittmatter SM (2009) Cellular prion protein mediates the toxicity of beta-amyloid oligomers: implications for Alzheimer disease. Arch Neurol 66: 1325–1328. 10.1001/archneurol.2009.223 19901162 PMC2849161

[15] Zhou J, Liu B (2013) Alzheimer's disease and prion protein. Intractable Rare Dis Res 2: 35–44. 10.5582/irdr.2013.v2.2.35 25343100 PMC4204584

[16] Prusiner SB (1998) Prions. Proc Natl Acad Sci U S A 95: 13363–13383. 9811807 10.1073/pnas.95.23.13363 PMC33918

[17] Prusiner SB (1982) Novel proteinaceous infectious particles cause scrapie. Science 216: 136–144. 6801762 10.1126/science.6801762

[18] Castellani RJ, Perry G, Smith MA (2004) Prion disease and Alzheimer's disease: pathogenic overlap. Acta Neurobiol Exp (Wars) 64: 11–17. 15190676 10.55782/ane-2004-1487

[19] Diedrich J, Wietgrefe S, Zupancic M, Staskus K, Retzel E, et al (1987) The molecular pathogenesis of astrogliosis in scrapie and Alzheimer's disease. Microb Pathog 2: 435–442. 3507557 10.1016/0882-4010(87)90050-7

[20] Checler F (2013) Alzheimer's and prion diseases: PDK1 at the crossroads. Nat Med 19: 1088–1090. 10.1038/nm.3332 24013743

[21] Jackson WS (2014) Selective vulnerability to neurodegenerative disease: the curious case of Prion Protein. Dis Model Mech 7: 21–29. 10.1242/dmm.012146 24396151 PMC3882045

[22] Jackson WS, Borkowski AW, Watson NE, King OD, Faas H, et al (2013) Profoundly different prion diseases in knock-in mice carrying single PrP codon substitutions associated with human diseases. Proc Natl Acad Sci U S A 110: 14759–14764. 10.1073/pnas.1312006110 23959875 PMC3767526

[23] Kovacs GG, Trabattoni G, Hainfellner JA, Ironside JW, Knight RS, et al (2002) Mutations of the prion protein gene phenotypic spectrum. J Neurol 249: 1567–1582. 12420099 10.1007/s00415-002-0896-9

[24] Bueler H, Aguzzi A, Sailer A, Greiner RA, Autenried P, et al (1993) Mice devoid of PrP are resistant to scrapie. Cell 73: 1339–1347. 8100741 10.1016/0092-8674(93)90360-3

[25] Weissmann C, Bueler H, Fischer M, Sailer A, Aguzzi A, et al (1994) PrP-deficient mice are resistant to scrapie. Ann N Y Acad Sci 724: 235–240. 8030944 10.1111/j.1749-6632.1994.tb38913.x

[26] Moore RC, Hope J, McBride PA, McConnell I, Selfridge J, et al (1998) Mice with gene targetted prion protein alterations show that Prnp, Sinc and Prni are congruent. Nat Genet 18: 118–125. 9462739 10.1038/ng0298-118

[27] Scott M, Groth D, Foster D, Torchia M, Yang SL, et al (1993) Propagation of prions with artificial properties in transgenic mice expressing chimeric PrP genes. Cell 73: 979–988. 8098995 10.1016/0092-8674(93)90275-u

[28] Biasini E, Turnbaugh JA, Unterberger U, Harris DA (2012) Prion protein at the crossroads of physiology and disease. Trends Neurosci 35: 92–103. 10.1016/j.tins.2011.10.002 22137337 PMC3273588

[29] Bouybayoune I, Mantovani S, Del Gallo F, Bertani I, Restelli E, et al (2015) Transgenic fatal familial insomnia mice indicate prion infectivity-independent mechanisms of pathogenesis and phenotypic expression of disease. PLoS Pathog 11: e1004796 10.1371/journal.ppat.1004796 25880443 PMC4400166

[30] Friedman-Levi Y, Meiner Z, Canello T, Frid K, Kovacs GG, et al (2011) Fatal prion disease in a mouse model of genetic E200K Creutzfeldt-Jakob disease. PLoS Pathog 7: e1002350 10.1371/journal.ppat.1002350 22072968 PMC3207931

[31] Hsiao KK, Scott M, Foster D, Groth DF, DeArmond SJ, et al (1990) Spontaneous neurodegeneration in transgenic mice with mutant prion protein. Science 250: 1587–1590. 1980379 10.1126/science.1980379

[32] Jackson WS, Borkowski AW, Faas H, Steele AD, King OD, et al (2009) Spontaneous generation of prion infectivity in fatal familial insomnia knockin mice. Neuron 63: 438–450. 10.1016/j.neuron.2009.07.026 19709627 PMC2775465

[33] Manson JC, Jamieson E, Baybutt H, Tuzi NL, Barron R, et al (1999) A single amino acid alteration (101L) introduced into murine PrP dramatically alters incubation time of transmissible spongiform encephalopathy. EMBO J 18: 6855–6864. 10581259 10.1093/emboj/18.23.6855 PMC1171748

[34] Jackson WS, Kaczmarczyk L (2015) Astonishing advances in mouse genetic tools for biomedical research. Swiss Med Wkly 145: w14186 10.4414/smw.2015.14186 26513700

[35] Bueler H, Fischer M, Lang Y, Bluethmann H, Lipp HP, et al (1992) Normal development and behaviour of mice lacking the neuronal cell-surface PrP protein. Nature 356: 577–582. 1373228 10.1038/356577a0

[36] Weissmann C, Bueler H (2004) A mouse to remember. Cell 116: S111–113, 112 p following S113. 15055597 10.1016/s0092-8674(04)00032-7

[37] Manson JC, Clarke AR, Hooper ML, Aitchison L, McConnell I, et al (1994) 129/Ola mice carrying a null mutation in PrP that abolishes mRNA production are developmentally normal. Mol Neurobiol 8: 121–127. 7999308 10.1007/BF02780662

[38] Moore RC, Redhead NJ, Selfridge J, Hope J, Manson JC, et al (1995) Double replacement gene targeting for the production of a series of mouse strains with different prion protein gene alterations. Biotechnology (N Y) 13: 999–1004. 9636277 10.1038/nbt0995-999

[39] Tuzi NL, Clarke AR, Bradford B, Aitchison L, Thomson V, et al (2004) Cre-loxP mediated control of PrP to study transmissible spongiform encephalopathy diseases. Genesis 40: 1–6. 15354287 10.1002/gene.20046

[40] Aymard F, Bugler B, Schmidt CK, Guillou E, Caron P, et al (2014) Transcriptionally active chromatin recruits homologous recombination at DNA double-strand breaks. Nat Struct Mol Biol 21: 366–374. 10.1038/nsmb.2796 24658350 PMC4300393

[41] Fritsch O, Benvenuto G, Bowler C, Molinier J, Hohn B (2004) The INO80 protein controls homologous recombination in Arabidopsis thaliana. Mol Cell 16: 479–485. 15525519 10.1016/j.molcel.2004.09.034

[42] Shaked H, Avivi-Ragolsky N, Levy AA (2006) Involvement of the Arabidopsis SWI2/SNF2 chromatin remodeling gene family in DNA damage response and recombination. Genetics 173: 985–994. 16547115 10.1534/genetics.105.051664 PMC1526515

[43] Fischer M, Rulicke T, Raeber A, Sailer A, Moser M, et al (1996) Prion protein (PrP) with amino-proximal deletions restoring susceptibility of PrP knockout mice to scrapie. Embo J 15: 1255–1264. 8635458 PMC450028

[44] Borchelt DR, Davis J, Fischer M, Lee MK, Slunt HH, et al (1996) A vector for expressing foreign genes in the brains and hearts of transgenic mice. Genet Anal 13: 159–163. 9117892 10.1016/s1050-3862(96)00167-2

[45] Yang W, Cook J, Rassbach B, Lemus A, DeArmond SJ, et al (2009) A New Transgenic Mouse Model of Gerstmann-Straussler-Scheinker Syndrome Caused by the A117V Mutation of PRNP. J Neurosci 29: 10072–10080. 10.1523/JNEUROSCI.2542-09.2009 19675240 PMC2749997

[46] Doudna JA, Charpentier E (2014) Genome editing. The new frontier of genome engineering with CRISPR-Cas9. Science 346: 1258096 10.1126/science.1258096 25430774

[47] Hsu PD, Lander ES, Zhang F (2014) Development and applications of CRISPR-Cas9 for genome engineering. Cell 157: 1262–1278. 10.1016/j.cell.2014.05.010 24906146 PMC4343198

[48] Qin W, Dion SL, Kutny PM, Zhang Y, Cheng A, et al (2015) Efficient CRISPR/Cas9-Mediated Genome Editing in Mice by Zygote Electroporation of Nuclease. Genetics. 10.1534/genetics.115.176594 PMC4492369 25819794

[49] Konermann S, Brigham MD, Trevino AE, Joung J, Abudayyeh OO, et al (2015) Genome-scale transcriptional activation by an engineered CRISPR-Cas9 complex. Nature 517: 583–588. 10.1038/nature14136 25494202 PMC4420636

[50] Eggan K, Rode A, Jentsch I, Samuel C, Hennek T, et al (2002) Male and female mice derived from the same embryonic stem cell clone by tetraploid embryo complementation. Nat Biotechnol 20: 455–459. 11981557 10.1038/nbt0502-455

[51] Nagy A (2003) Manipulating the mouse embryo: a laboratory manual New York: Cold Spring Harbor Laboratory Press x, 764 p. p.

[52] Stacey A, Schnieke A, McWhir J, Cooper J, Colman A, et al (1994) Use of double-replacement gene targeting to replace the murine alpha-lactalbumin gene with its human counterpart in embryonic stem cells and mice. Mol Cell Biol 14: 1009–1016. 8289781 10.1128/mcb.14.2.1009-1016.1994 PMC358456

[53] Jackson WS, Krost C, Borkowski AW, Kaczmarczyk L (2014) Translation of the prion protein mRNA is robust in astrocytes but does not amplify during reactive astrocytosis in the mouse brain. PLoS One 9: e95958 10.1371/journal.pone.0095958 24752288 PMC3994155

[54] Ran FA, Hsu PD, Wright J, Agarwala V, Scott DA, et al (2013) Genome engineering using the CRISPR-Cas9 system. Nat Protoc 8: 2281–2308. 10.1038/nprot.2013.143 24157548 PMC3969860

[55] Gilbert LA, Larson MH, Morsut L, Liu Z, Brar GA, et al (2013) CRISPR-mediated modular RNA-guided regulation of transcription in eukaryotes. Cell 154: 442–451. 10.1016/j.cell.2013.06.044 23849981 PMC3770145

[56] Heintze J, Luft C, Ketteler R (2013) A CRISPR CASe for high-throughput silencing. Front Genet 4: 193 10.3389/fgene.2013.00193 24109485 PMC3791873

[57] Doench JG, Hartenian E, Graham DB, Tothova Z, Hegde M, et al (2014) Rational design of highly active sgRNAs for CRISPR-Cas9-mediated gene inactivation. Nat Biotechnol 32: 1262–1267. 10.1038/nbt.3026 25184501 PMC4262738

[58] Priola SA, McNally KL (2009) The role of the prion protein membrane anchor in prion infection. Prion 3: 134–138. 19786843 10.4161/pri.3.3.9771 PMC2802777

[59] Aguzzi A, Rajendran L (2009) The transcellular spread of cytosolic amyloids, prions, and prionoids. Neuron 64: 783–790. 10.1016/j.neuron.2009.12.016 20064386

[60] Cushman M, Johnson BS, King OD, Gitler AD, Shorter J (2010) Prion-like disorders: blurring the divide between transmissibility and infectivity. J Cell Sci 123: 1191–1201. 10.1242/jcs.051672 20356930 PMC2848109

[61] Saxena S, Caroni P (2011) Selective neuronal vulnerability in neurodegenerative diseases: from stressor thresholds to degeneration. Neuron 71: 35–48. 10.1016/j.neuron.2011.06.031 21745636

[62] Salmena L, Poliseno L, Tay Y, Kats L, Pandolfi PP (2011) A ceRNA hypothesis: the Rosetta Stone of a hidden RNA language? Cell 146: 353–358. 10.1016/j.cell.2011.07.014 21802130 PMC3235919

[63] Tay Y, Rinn J, Pandolfi PP (2014) The multilayered complexity of ceRNA crosstalk and competition. Nature 505: 344–352. 10.1038/nature12986 24429633 PMC4113481

[64] Mehrabian M, Brethour D, MacIsaac S, Kim JK, Gunawardana CG, et al (2014) CRISPR-Cas9-based knockout of the prion protein and its effect on the proteome. PLoS One 9: e114594 10.1371/journal.pone.0114594 25490046 PMC4260877

[65] Baybutt H, Manson J (1997) Characterisation of two promoters for prion protein (PrP) gene expression in neuronal cells. Gene 184: 125–131. 9016962 10.1016/s0378-1119(96)00600-2

[66] Ran FA, Hsu PD, Lin CY, Gootenberg JS, Konermann S, et al (2013) Double nicking by RNA-guided CRISPR Cas9 for enhanced genome editing specificity. Cell 154: 1380–1389. 10.1016/j.cell.2013.08.021 23992846 PMC3856256

[67] Maruyama T, Dougan SK, Truttmann MC, Bilate AM, Ingram JR, et al (2015) Increasing the efficiency of precise genome editing with CRISPR-Cas9 by inhibition of nonhomologous end joining. Nat Biotechnol 33: 538–542. 10.1038/nbt.3190 25798939 PMC4618510

[68] Cho SW, Kim S, Kim Y, Kweon J, Kim HS, et al (2014) Analysis of off-target effects of CRISPR/Cas-derived RNA-guided endonucleases and nickases. Genome Res 24: 132–141. 10.1101/gr.162339.113 24253446 PMC3875854

[69] Heigwer F, Kerr G, Boutros M (2014) E-CRISP: fast CRISPR target site identification. Nat Methods 11: 122–123. 10.1038/nmeth.2812 24481216

[70] Kleinstiver BP, Pattanayak V, Prew MS, Tsai SQ, Nguyen NT, et al (2016) High-fidelity CRISPR-Cas9 nucleases with no detectable genome-wide off-target effects. Nature 529: 490–495. 10.1038/nature16526 26735016 PMC4851738

[71] Wang H, Yang H, Shivalila CS, Dawlaty MM, Cheng AW, et al (2013) One-step generation of mice carrying mutations in multiple genes by CRISPR/Cas-mediated genome engineering. Cell 153: 910–918. 10.1016/j.cell.2013.04.025 23643243 PMC3969854

[72] Remy S, Tesson L, Menoret S, Usal C, De Cian A, et al (2014) Efficient gene targeting by homology-directed repair in rat zygotes using TALE nucleases. Genome Res 24: 1371–1383. 10.1101/gr.171538.113 24989021 PMC4120090

[73] Yang H, Wang H, Shivalila CS, Cheng AW, Shi L, et al (2013) One-step generation of mice carrying reporter and conditional alleles by CRISPR/Cas-mediated genome engineering. Cell 154: 1370–1379. 10.1016/j.cell.2013.08.022 23992847 PMC3961003

[74] Dametto P, Lakkaraju AK, Bridel C, Villiger L, O'Connor T, et al (2015) Neurodegeneration and unfolded-protein response in mice expressing a membrane-tethered flexible tail of PrP. PLoS One 10: e0117412 10.1371/journal.pone.0117412 25658480 PMC4319788

[75] Herrmann US, Sonati T, Falsig J, Reimann RR, Dametto P, et al (2015) Prion infections and anti-PrP antibodies trigger converging neurotoxic pathways. PLoS Pathog 11: e1004662 10.1371/journal.ppat.1004662 25710374 PMC4339193

[76] Kurt TD, Bett C, Fernandez-Borges N, Joshi-Barr S, Hornemann S, et al (2014) Prion transmission prevented by modifying the beta2-alpha2 loop structure of host PrPC. J Neurosci 34: 1022–1027. 10.1523/JNEUROSCI.4636-13.2014 24431459 PMC3891945

[77] Sigurdson CJ, Nilsson KP, Hornemann S, Heikenwalder M, Manco G, et al (2009) De novo generation of a transmissible spongiform encephalopathy by mouse transgenesis. Proc Natl Acad Sci U S A 106: 304–309. 10.1073/pnas.0810680105 19073920 PMC2629180

[78] Yewdell JW (2001) Not such a dismal science: the economics of protein synthesis, folding, degradation and antigen processing. Trends Cell Biol 11: 294–297. 11413040 10.1016/s0962-8924(01)02030-x

[79] Harris DA (2003) Trafficking, turnover and membrane topology of PrP. Br Med Bull 66: 71–85. 14522850 10.1093/bmb/66.1.71

[80] Caughey B, Race RE, Ernst D, Buchmeier MJ, Chesebro B (1989) Prion protein biosynthesis in scrapie-infected and uninfected neuroblastoma cells. J Virol 63: 175–181. 2562814 10.1128/jvi.63.1.175-181.1989 PMC247670

[81] Kocisko DA, Baron GS, Rubenstein R, Chen J, Kuizon S, et al (2003) New inhibitors of scrapie-associated prion protein formation in a library of 2000 drugs and natural products. J Virol 77: 10288–10294. 12970413 10.1128/JVI.77.19.10288-10294.2003 PMC228499

[82] Butler DA, Scott MR, Bockman JM, Borchelt DR, Taraboulos A, et al (1988) Scrapie-infected murine neuroblastoma cells produce protease-resistant prion proteins. J Virol 62: 1558–1564. 3282080 10.1128/jvi.62.5.1558-1564.1988 PMC253182

[83] Falsig J, Aguzzi A (2008) The prion organotypic slice culture assay—POSCA. Nat Protoc 3: 555–562. 10.1038/nprot.2008.13 18388937

[84] Falsig J, Sonati T, Herrmann US, Saban D, Li B, et al (2012) Prion pathogenesis is faithfully reproduced in cerebellar organotypic slice cultures. PLoS Pathog 8: e1002985 10.1371/journal.ppat.1002985 23133383 PMC3486912

[85] Sonati T, Reimann RR, Falsig J, Baral PK, O'Connor T, et al (2013) The toxicity of antiprion antibodies is mediated by the flexible tail of the prion protein. Nature 501: 102–106. 10.1038/nature12402 23903654

