# Supplementary material for: Manipulating the Prion Protein Gene Sequence and Expression Levels with CRISPR/Cas9
Source: PLoS One. 2016 Apr 29;11(4):e0154604. doi: 10.1371/journal.pone.0154604 (PMC4851410; doi:10.1371/journal.pone.0154604)
Supplement: S1 Fig — (PDF) [file pone.0154604.s001.pdf]

Figure S1

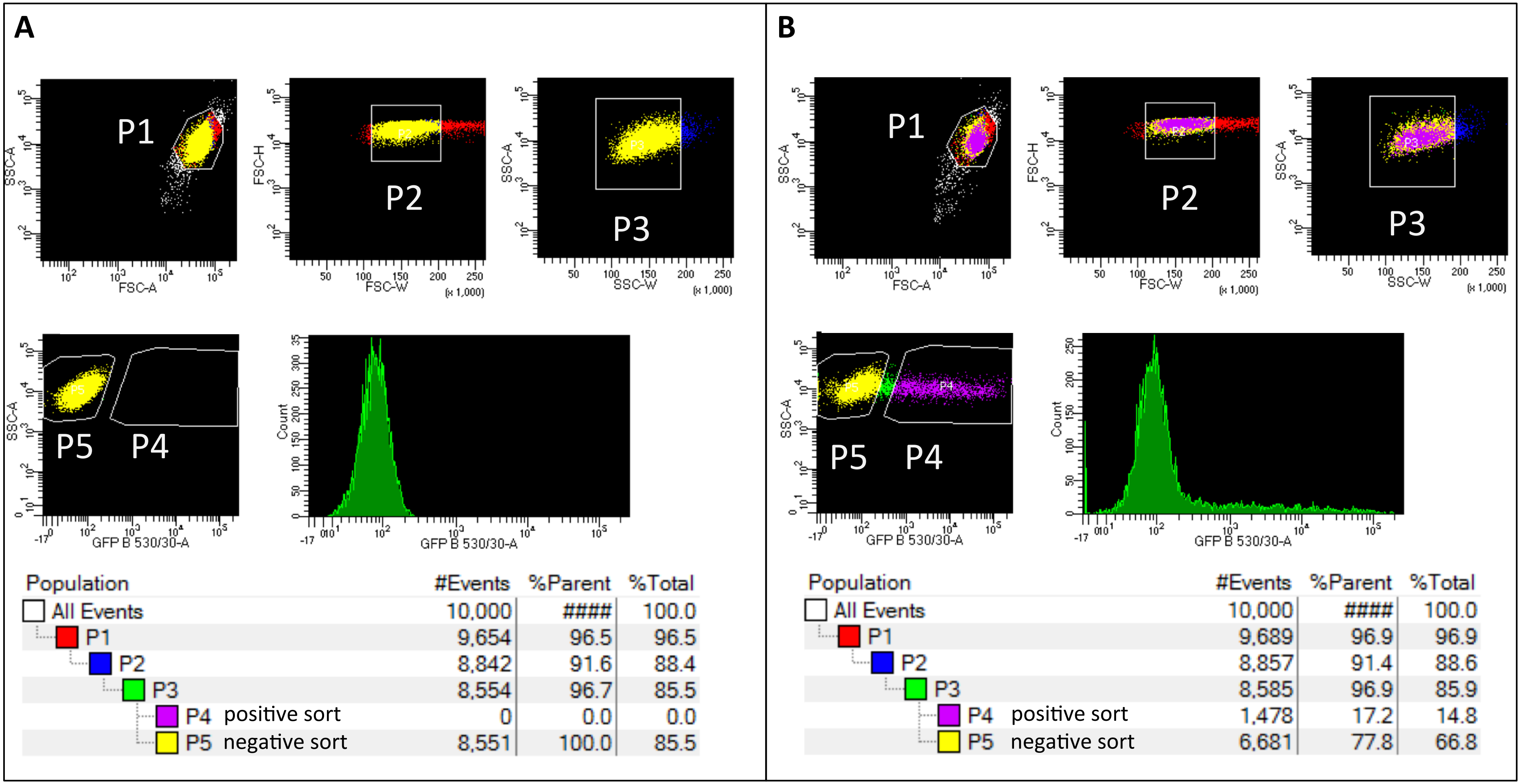

S1 Fig. **FACS sorting of N2a cells.** Plots for representative positive sample and negative control were shown.
