# Supplementary material for: Manipulating the Prion Protein Gene Sequence and Expression Levels with CRISPR/Cas9
Source: PLoS One. 2016 Apr 29;11(4):e0154604. doi: 10.1371/journal.pone.0154604 (PMC4851410; doi:10.1371/journal.pone.0154604)
Supplement: S1 Table — (PDF) [file pone.0154604.s002.pdf]

Position on the scheme (Fig 2A)	Primer name	Primer sequence (5'-3')	Function
p5	pBS_KS(+)_F	GATAGTTGCTGAGCGTCGTCA	pBluseScript forward primer used for verification of absence of vector backbone after homologous recombination
p6	pBS_KS(+)_R	GGGAGTGCTGACACTGGGGG	pBluseScript reverse primer used for verification of absence of vector backbone after homologous recombination
p1	Prnp_genomic_F	GAAGGGAAGTTCTGGCTGCT	Forward genomic primer upstream of left homology arm
p4	Prnp_genomic_R	TCACCCACACCCTCCACAT	Reverse genomic primer downstream of right homology arm
p2	Prnp_BV&MS_R	GCCCCCGCGAGAGACGCGGCG	Common reverse primer for Bank Vole and Mouse PrP coding sequence
p3a	Prnp_BV_F	GCTAGGCTGGGCGAGGGGCG	Forward primer for Bank Vole PrP coding sequence
p3b	EGFP_F	CCAACGAGAAGCGCGATCAC	Forward primer for EGFP coding sequence (used to genotype PrP-EGFP fusion transgene)

Table S1. **Primer sequences used for genotyping ES cell clones.**
