# Supplementary material for: Manipulating the Prion Protein Gene Sequence and Expression Levels with CRISPR/Cas9
Source: PLoS One. 2016 Apr 29;11(4):e0154604. doi: 10.1371/journal.pone.0154604 (PMC4851410; doi:10.1371/journal.pone.0154604)
Supplement: S2 Table — (PDF) [file pone.0154604.s003.pdf]

| Guide name | Spacer sequence | PAM | Strand | Region targeted | sgRNA score [ref. 57] |
|---|---|---|---|---|---|
| HR Prnp 1 | GCCCGGGATACCGGCTTCCAC | CGG | antisense | 103 nucleotide of ORF | 0.31 |
| HR Prnp 2 | GGATCTTCTCCCGTCGTAAT | AGG | antisense | 673 nucleotide of ORF | 0.21 |
| HR Prnp 3 | GCGAGACCGATGTGAAGATGA | TGG | sense | 610 nucleotide of ORF | 0.16 |
| HR Prnp 4 | GATATTGCATGGTTGTTACGCCA | TGG | antisense | 255 nucleotide upstream of exon 3 | 0.18 |
| HR Prnp 5 | GACCAGACGTGGTTTACCAGT | TGG | sense | 212 nucleotide upstream of exon 3 | 0.22 |
| HR Prnp 6 | GAACCTTGGCTACTGGCTGC | TGG | sense | 33 nucleotide of ORF | 0.01 |

S2 Table. **CC9 guide sequences used to stimulate HR in Prnp locus.** PAM – protospacer adjacent motif.
