# Supplementary material for: Manipulating the Prion Protein Gene Sequence and Expression Levels with CRISPR/Cas9
Source: PLoS One. 2016 Apr 29;11(4):e0154604. doi: 10.1371/journal.pone.0154604 (PMC4851410; doi:10.1371/journal.pone.0154604)
Supplement: S3 Table — (PDF) [file pone.0154604.s004.pdf]

Guide name	Spacer sequence	PAM	Strand	Distance from TSS	sgRNA score [ref. 57]	Found by design SAM sgRNA prediction tool (http://sam.genome-engineering.org)
Prnp SAM 1	GATAGTTGCTGAGCGTCGTCA	GGG	sense	TSS -158	0.79	no
Prnp SAM 2	GGGAGTGCTGACACTGGGGG	CGG	sense	TSS -138	0.67	yes
Prnp SAM 3	GCATTTAAGCCAGTCCGGAG	CGG	sense	TSS -100	0.63	no
Prnp SAM 4	GTATAGTTGCTGAGCGTCGTC	AGG	sense	TSS -159	0.17	yes
Prnp SAM 5	GCCACCCCCCCGCGAGAGACG	CGG	sense	TSS -58	0.62	no
Prnp SAM 6	GCCCCCGCGAGAGACGCGGCG	CGG	sense	TSS -53	0.60	no
Prnp SAM 7	GCTAGGCTGGGCGAGGGGCG	GGG	antisense	TSS -29	0.26	no
Prnp SAM 8	GCCGCGCCGCGTCTCTCGCG	GGG	antisense	TSS -68	0.25	no
Prnp SAM 9	GCGGGGCGTGATGCTCACCAA	TGG	antisense	TSS -47	0.12	yes

Table S3. **Guide sequences used to generate SAM sgRNAs for activating Prnp gene.** TSS – transcription start site; PAM – protospacer adjacent motif.
