# Supplementary material for: Manipulating the Prion Protein Gene Sequence and Expression Levels with CRISPR/Cas9
Source: PLoS One. 2016 Apr 29;11(4):e0154604. doi: 10.1371/journal.pone.0154604 (PMC4851410; doi:10.1371/journal.pone.0154604)
Supplement: S4 Table — (PDF) [file pone.0154604.s005.pdf]

Figure 2C raw data

	Experiment 1			Experiment 2			Experiment 3		
	clones analyzed	clones positive	%	clones analyzed	clones positive	%	clones analyzed	clones positive	%
no CC9	10	0	**0%**	8	1	**13%**	8	1	**13%**
HR Prnp 1	11	2	**18%**	8	1	**13%**	8	2	**25%**
HR Prnp 2	11	0	**0%**	8	3	**38%**	8	2	**25%**
HR Prnp 3	11	7	**64%**	8	6	**75%**	8	5	**63%**
HR Prnp 4	9	6	**67%**	8	4	**50%**	8	4	**50%**
HR Prnp 5	11	3	**27%**	8	3	**38%**	8	4	**50%**
HR Prnp 6	10	2	**20%**	8	1	**13%**	8	2	**25%**

Figure 3B raw data

	Experiment 1			Experiment 2			Experiment 3		
	clones analyzed	clones positive	%	clones analyzed	clones positive	%	clones analyzed	clones positive	%
no CC9	12	1	**8%**	8	1	**13%**	12	1	**8%**
HR Prnp 3	12	11	**92%**	12	10	**83%**	11	10	**91%**
Prnp (WT/HPRT) + HR Prnp 3	8	6	**75%**	8	7	**88%**	8	6	**75%**

	Experiment 1			Experiment 2			Experiment 3		
	clones analyzed	clones positive	%	clones analyzed	clones positive	%	clones analyzed	clones positive	%
no CC9	8	6	**75%**	12	11	**92%**	12	1	**8%**
HR Prnp 3	8	7	**88%**	12	10	**83%**	8	1	**13%**
Prnp (WT/HPRT) + HR Prnp 3	8	6	**75%**	11	10	**91%**	12	1	**8%**

Figure 4C raw data

	**Ct values**				**Houskeeper avg**		**Prnp_assay1**			**Prnp_assay2**		
	ACTB	HPRT	Prnp_assay1	Prnp_assay2	avg Ct	dCt (vs. no sgRNA	dCt (vs. no sgRNA	ddCt	Increment	dCt (vs. no sgRNA)	ddCt	Increment
**no sgRNA2.0**	**22.55**	**26.91**	**27.84**	**27.42**	**24.73**	**-1.85**	**-1.26**	**-0.59**	**66%**	**-1.69**	**-0.16**	**89%**
Prnp SAM1	23.81	28.31	24.94	24.75	26.06	-0.52	-4.16	3.64	1242%	-4.35	3.82	1417%
Prnp SAM2	24.27	28.88	25.55	25.38	26.57	-0.01	-3.55	3.54	1165%	-3.72	3.71	1309%
Prnp SAM3	23.62	27.64	25.55	25.14	25.63	-0.95	-3.54	2.59	602%	-3.96	3.01	805%
Prnp SAM4	24.02	28.41	26.69	26.38	26.22	-0.37	-2.41	2.04	411%	-2.72	2.36	512%
Prnp SAM5	23.50	27.97	25.07	24.90	25.74	-0.85	-4.03	3.18	907%	-4.20	3.35	1022%
Prnp SAM6	25.09	29.13	26.56	26.55	27.11	0.53	-2.54	3.07	838%	-2.55	3.08	847%
Prnp SAM7	23.74	27.53	26.86	26.89	25.63	-0.95	-2.23	1.28	244%	-2.21	1.26	240%
Prnp SAM8	24.73	28.58	27.60	27.33	26.66	0.08	-1.50	1.57	298%	-1.77	1.84	358%
Prnp SAM9	24.85	28.70	26.14	26.39	26.77	0.19	-2.95	3.14	883%	-2.71	2.90	748%
**Untreated cells**	**24.49**	**28.68**	**29.09**	**29.10**	**26.58**	**0.00**	**0.00**	**0.00**	**100%**	**0.00**	**0.00**	**100%**

S4 Table. Gene targeting experiments and SAM screening – raw data.
